# Is Urology a gender-biased career choice? A survey-based study of the Italian medical students' perception of specialties

**DOI:** 10.3389/fsurg.2022.962824

**Published:** 2022-07-29

**Authors:** Sofia Reale, Luca Orecchia, Simona Ippoliti, Simone Pletto, Serena Pastore, Stefano Germani, Alessandra Nardi, Roberto Miano

**Affiliations:** ^1^ Urology Unit, CHUV – Centre Hospitalier Universitaire Vaudois, Lausanne, Switzerland; ^2^ Urology Unit, Policlinico Tor Vergata Foundation, Rome, Italy; ^3^ Urology Department, Harrogate and District NHS Foundation Trust, Harrogate, United Kingdom; ^4^ Department of Mathematics, University of Rome Tor Vergata, Rome, Italy; ^5^ Department of Surgical Sciences, University of Rome Tor Vergata, Rome, Italy

**Keywords:** Feminisation of medicine, specialty training, urology training, medical students, sexist environment

## Abstract

**Background:**

Despite the well-established worldwide phenomenon of “the feminisation of medicine,” in Italy, Urology remains a male-dominated field.

**Objective:**

The aims of our work are to assess data on medical students' choice of surgical specialty in Italy to investigate if a gender-biased trend exists and to find the key points that influence the decision-making process when choosing a specialty, with a focus on Urology.

**Design:**

Data about access to residency programs in 2017–2020 were analysed through descriptive statistics. Investigations concerning the decision-making process were carried through distribution of an online anonymous survey to Italian medical students.

**Results:**

Urology was among the specialties with the lowest proportion of female residents in Italy in the last 4 years: 37 (29.4%) in 2017, 27 (21.4%) in 2018, 40 (26.7%) in 2019, and 57 (25.2%) in 2020. The total number of participants of the survey was 1409, of which only 341 declared being keen to pursue a career path in surgery. Out of the 942 students not interested in surgery, 46.2% females and 22.5% males indicated a “sexist environment” as one of the reasons. Overall, the main reason for medical students not choosing Urology is the lack of interest in the specialty. Furthermore, there is a different perception of Urology as a sexist environment between female (23.4%) and male (3.2%, *p* < 0.001) medical students, which may influence their decision-making process.

**Conclusions:**

In Italy, the prevalence of female medical graduates does not mirror the proportion of female doctors choosing a career in some surgical specialties, including Urology. Our survey results clearly identified that a large proportion of medical graduates are not choosing urology because of the perception of a sexist environment. While the reasons for this phenomenon remain unclear, the presence of a gender-biased perception of a sexist environment represents a possible explanation.

## Introduction

Over the past century, women have moved from legal exclusion from medical schools to accounting for the majority of medical school applicants and graduates, a trend that has been referred to as “the feminisation of medicine” (1).

In particular, since the beginning of the 21st century, an increasingly high number of women have been choosing a career in medicine, and as a result, the number of female medical students equals or exceeds that of male medical students in several industrialised countries worldwide, including France (2), United Kingdom (3), Spain (4), Germany (5), United States (6), Canada (7), Israel (8), and Italy (9).

Although women are increasingly taking to the medical profession, some skews persist as far as gender distribution among specialties is concerned. In fact, some medical specialties, including General Surgery and Orthopaedics, are significantly male dominated, while others are more female dominated, such as Gynaecology and Paediatrics (10).

Urology has historically been a field dominated by male physicians (11) and, despite the general trend of medical feminisation in the 21st century, it seems to be attracting primarily male applicants, and the search for the reasons of this specialty being rarely chosen by women should explore different domains.

Despite these hindrances, Urology remains a competitive surgical specialty; therefore, understanding the factors affecting students' overall consideration of Urology as a career is an important step to develop strategies aimed at ensuring that this field continues to attract excellent candidates (12).

In Italy, access to residency programs is regulated by a multiple-choice national test. Participants are ranked into one single national ranking that establishes the priority of each candidate to enrol into a residency program of their choice, until all places in every residency program are allocated. The Italian Ministry of Education subdivides residency programs into three areas: medical, surgical (including Urology), and services. For the detailed list of specialties pertaining to each area, refer to Supplementary Table S2.

The aims of our work are: (1) to assess data on the choice of surgical specialty in Italy to investigate if a gender-biased trend exists and (2) to identify the key points that influence the decision-making process when choosing the specialty, with a particular focus on Urology.

## Materials and methods

### 2017–2020 Italian trends

Data about access to residency programs in the Year 2017–2020 were analysed from a dedicated database provided by the Associazione Liberi Specializzandi (ALS) Association.

Extracted data pertaining to 2017–2020 were divided by year, gender, and area of specialty chosen (Medical, Surgical, and Services). The surgical area was further analysed and subdivided by each available single surgical specialty: General surgery, Paediatric surgery, Plastic surgery, Obstetrics and Gynaecology, Orthopaedics, Urology, Maxillofacial surgery, Neurosurgery, Ophthalmology, ENT, Cardiac surgery, Thoracic surgery, and Vascular surgery.

Data extraction was done by two authors (AN, SR) and then cross-checked by a lead researcher (RM).

### Survey

A completely anonymous electronic survey was designed on the platform SurveyMonkey and was distributed in 2019 for 30 days through social media platforms.

The survey was targeted at students who were approaching their specialty training and were enrolled into the fourth, fifth, and sixth year of medical school in 2019 in Italy.

**Figure 1:**
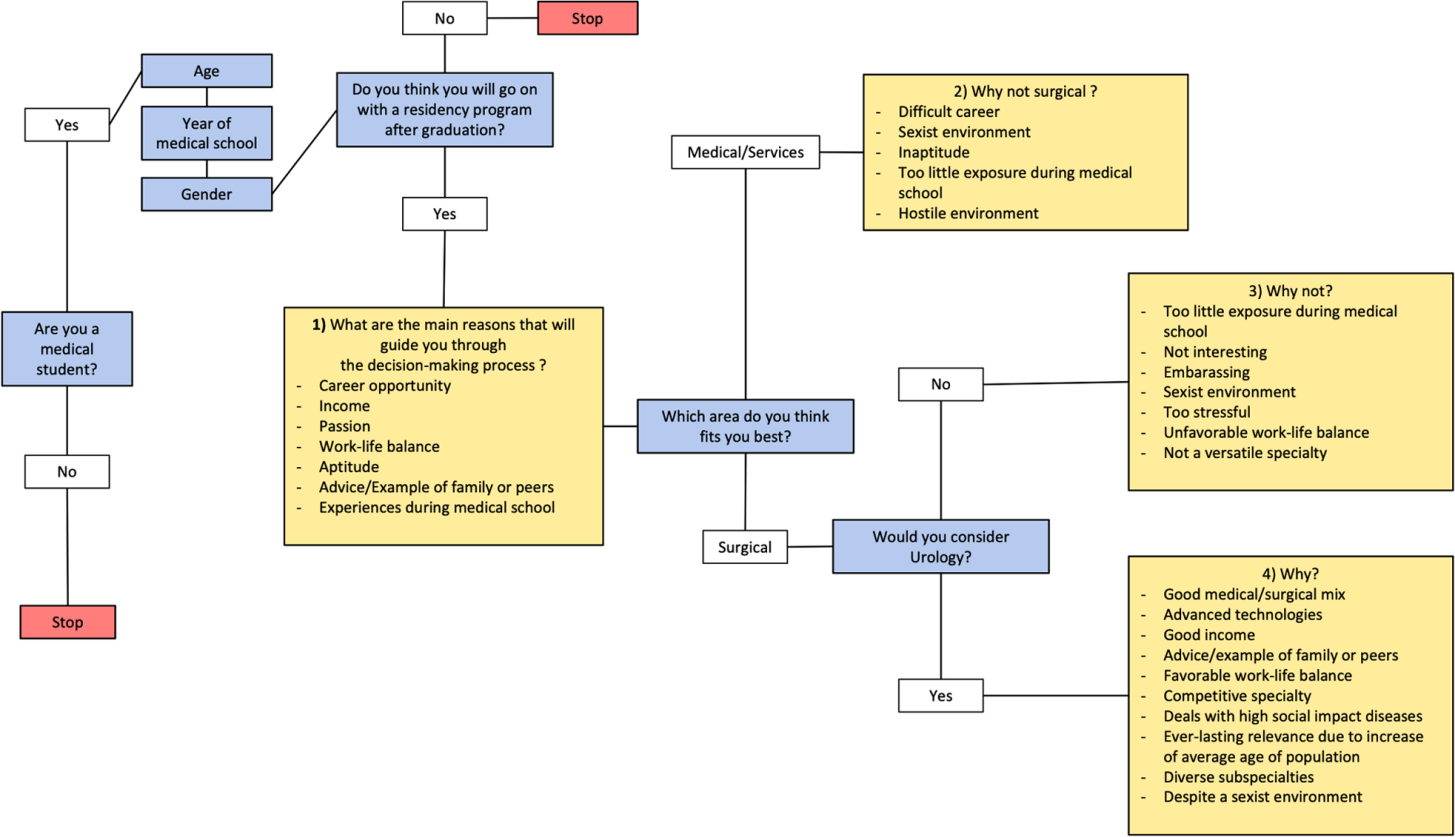
Overview of a questionnaire structure, with multiple-choice questions given in blue and Likert-scale questions given in yellow.

The survey consisted of five to nine total questions for each respondent. Content validity and comprehensiveness were verified before commencement of the study by piloting among interns, medical students, and university lecturers. The number of questions varied due to the presence of two multiple-choice questions whose negative response led to the termination of the questionnaire, excluding participants who would not be relevant to the investigation. There were two question types: multiple-choice questions and 1–5 Likert-scale questions (1 = strongly disagree – 5 = strongly agree). The complete survey structure is presented in Figure 1.

### Data analysis

Continuous variables were summarised by mean and standard deviation; categorical variables were described by absolute and relative frequencies.

The association between two categorical variables was evaluated by using the Chi-square test; Fisher exact test was preferred in case of sparse tables. Continuous covariates were compared by using the *t*-test or Wilcoxon rank-sum test when a significant departure from normality was detected.

## Results

### 2017–2020 Trends

Between 2017 and 2020, the percentage of females graduating from medical school remained substantially stable (55.5% in 2017, 53.9% in 2018, 55.3% in 2019, and 55.8% in 2020), showing that every year most of the graduates were females.

This is reflected in the overall gender distribution of matriculants to a specialty program in the same year intervals, where female predominance over male is evident (female matriculants: 57.1% in 2017, 56.2% in 2018, 53.9% in 2019, and 56.3% in 2020).

**Figure 2:**
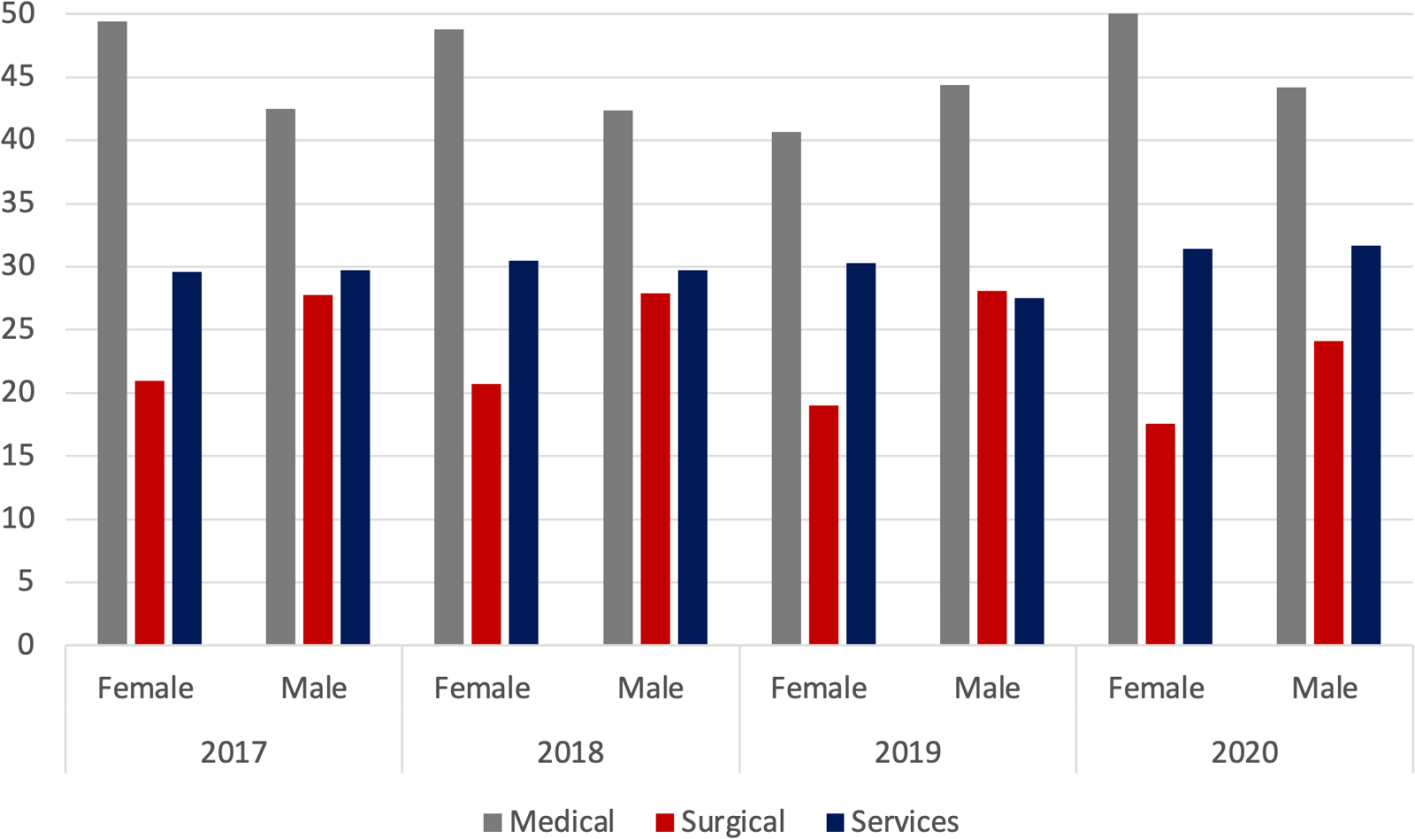
Gender distribution among the three areas in percentage: medical, surgical, services.

Gender distributions of matriculants in the three areas (medical, services, and surgical) showed that in 2020, of all female matriculants, 51.0% were in the medical area, 31.4% in the services area, and 17.6% in the surgical area, whereas of all male matriculants, 44.2% were in the medical area, 31.7% in the services area, and 24.1% in the surgical area (Figure 2). Moreover, considering only matriculants in the surgical area, a slight decrease in the number of females from 50.2% in 2017 to 48.4% in 2020 was evidenced (Table 1).

**Table 1:** Matriculants into the three areas in the years 2017–2020, with the percentage of female matriculants in each area shown in brackets.

	2017	2018	2019	2020
Surgical (F%)	1,635 (50.2)	1,667 (48.8)	2,017 (44.2)	2,977 (48.4)
Medical (F%)	3,165 (60.7)	3,221 (59.6)	4,150 (57.2)	6,999 (59.8)
Services (F%)	2,021 (57.0)	2,110 (56.8)	2,517 (56.3)	4,608 (56.1)

**Figure 3:**
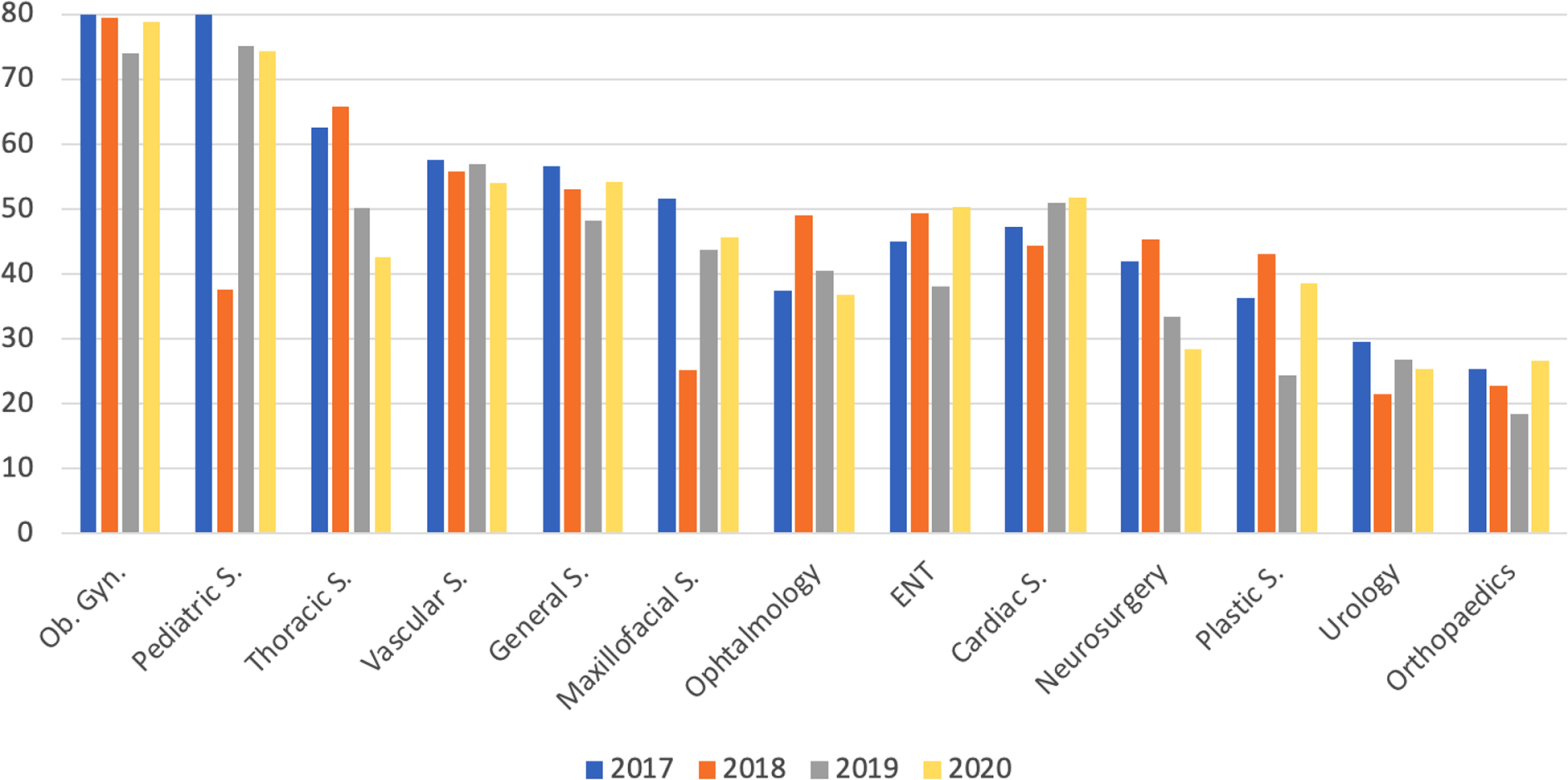
Percentage of candidates matriculating into a surgical residency program that were female, in the 2017–2020 period.

The percentage of female matriculants in all the surgical specialties during the 2017–2020-year interval is shown in (Figure 3). Urology was among the specialities with the lowest proportion of females: 37 (29.4%) in 2017, 27 (21.4%) in 2018, 40 (26.7%) in 2019, and 57 (25.2%) in 2020.

### Survey

**Figure 4:**
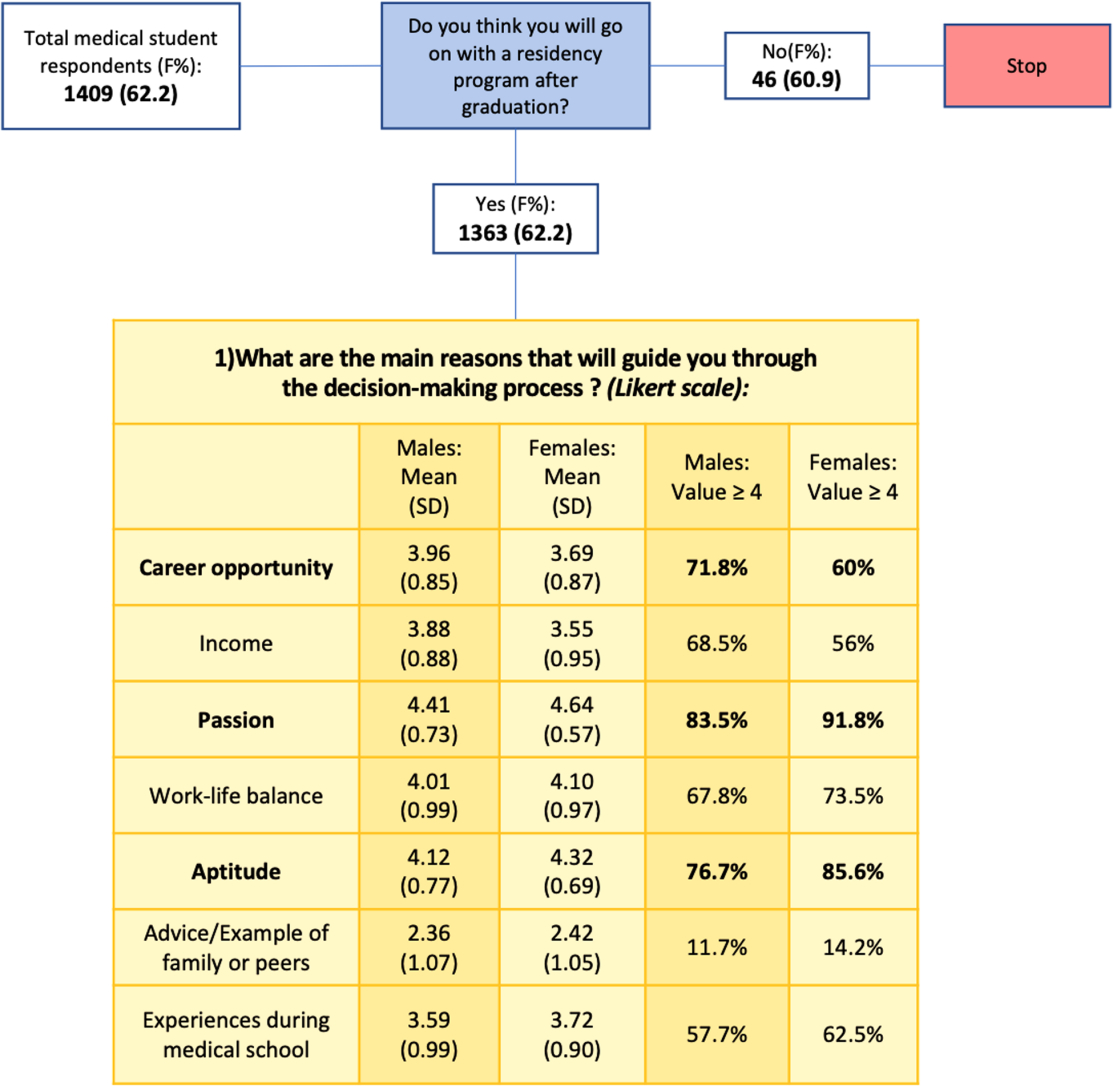
Reasons guiding the decision-making process of a program, with male and female respondents expressed as mean, standard deviation (SD), and values ≥4.

The total number of medical student participants in the survey was 1409. Gender distribution of respondents was 62.1% females and 37.9% males. A total of 1363 medical students responded fully to the first set of predefined five-point Likert scale answers about the main motives and reasons guiding the process of choosing a residency program. The overall main reason indicated by both genders was “Passion”, with a mean of 4.64 for female and 4.41 male respondents. Meanwhile, the percentage of value ≥4 for female respondents was the highest for “Passion” and “Attitude”, while for male respondents, it was the highest for “Career opportunity” (Figure 4).

**Figure 5:**
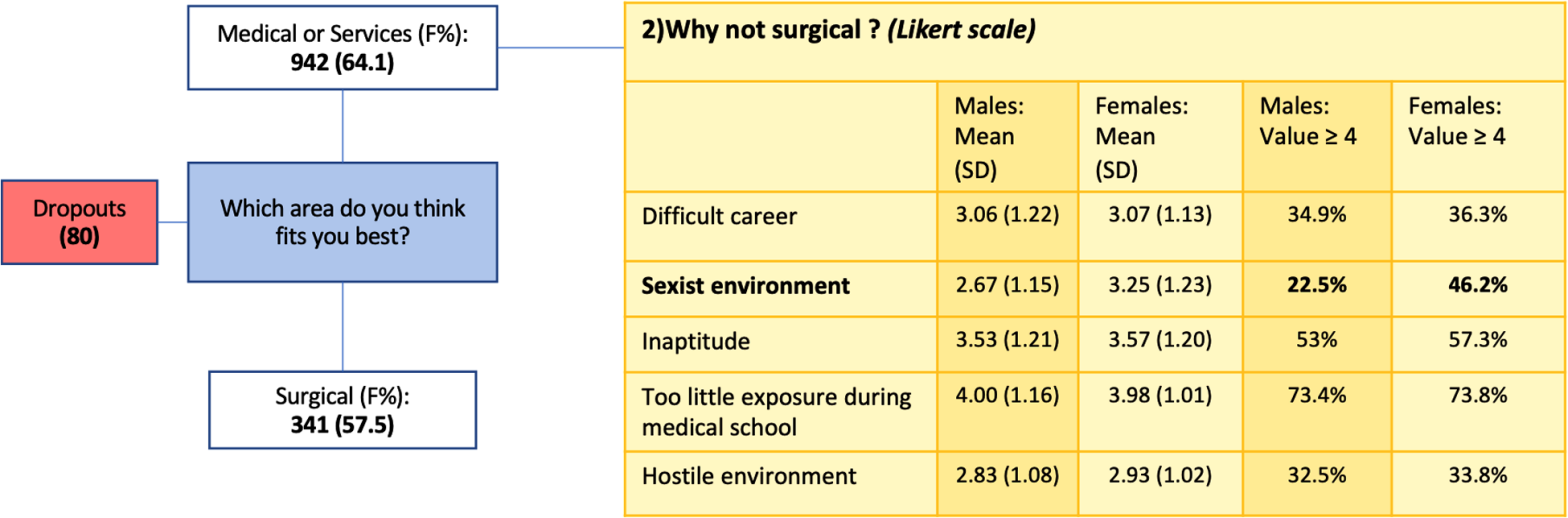
Reasons for not choosing a surgical program, with male and female respondents expressed as mean, standard deviation (SD), and values ≥4. Dropouts were respondents who did not answer all the questions of the survey.

A total of 942 students, corresponding to 69.1% of all respondents, declared to be keen to choose a specialty pertaining to the Medical or Services groups. These students were then provided with a list of five statements concerning the reasons behind their propensity to avoid surgical specialties, where the main reason indicated was “Too little exposure during studies,” accounting for a mean of about four for both genders. The values assigned to each statement were almost equal for both female and male respondents, except for “Sexist environment” with a statistically significant difference in percentage of value ≥4, 22.5% for males and 46.2% for females (*p* < 0.0001) (Figure 5).

A total of 341 students who declared being keen to pursue a career path in surgery were then sorted between 236 (69.2%) who denied considering Urology as a possible specialty and 105 (30.8%) who confirmed including Urology among their specialties of choice.

**Figure 6:**
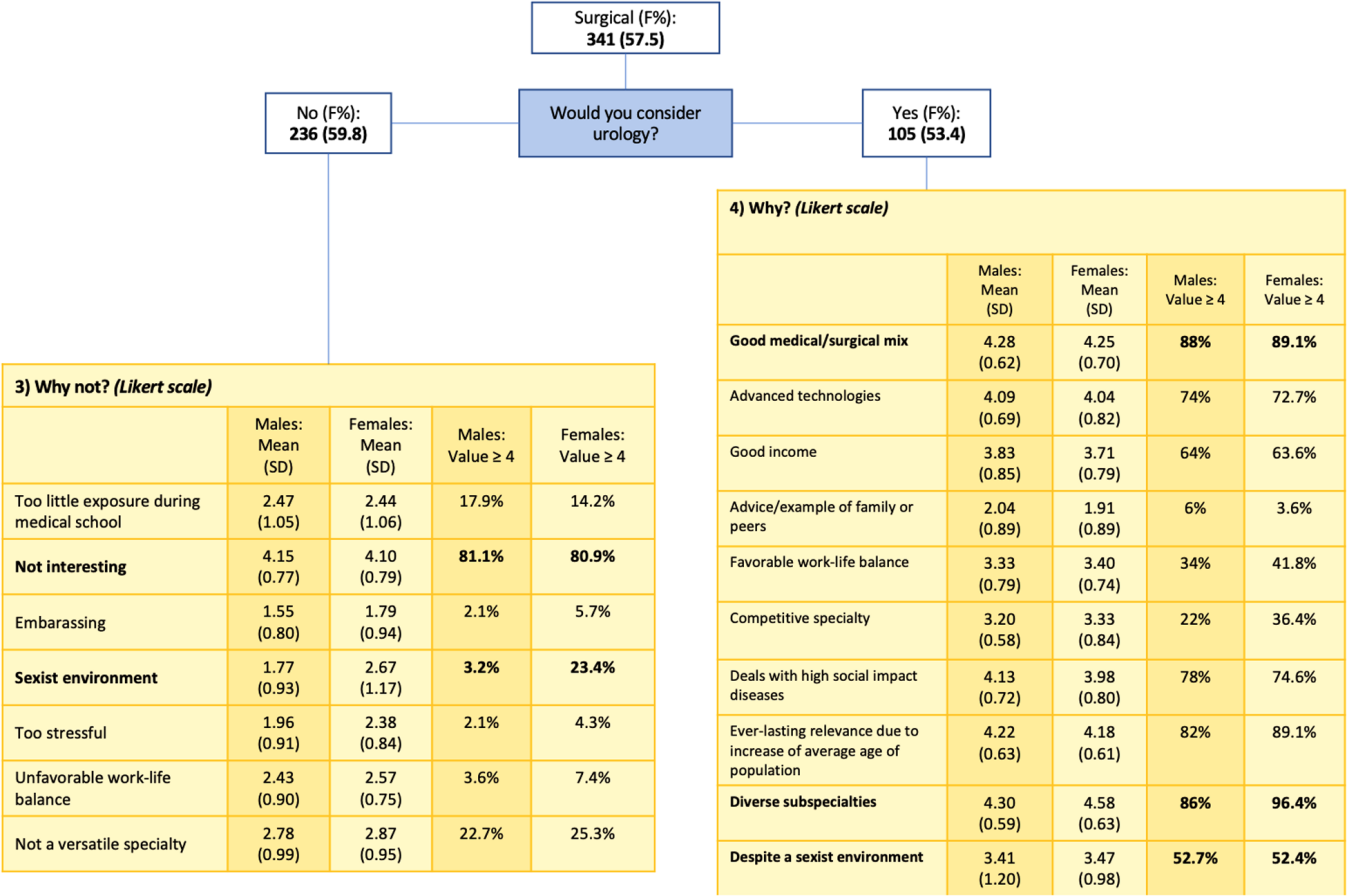
Reasons for not choosing vs. choosing Urology, with male and female respondents expressed as mean, standard deviation (SD), and values ≥4.

Among those who declared considering Urology as a choice, the main reasons indicated were “Diverse subspecialties” and “Good medical/surgical mix.” Surprisingly, male and female respondents attributed a value ≥4 in similar percentages to all the statements, including the one concerning the perception of a sexist environment in Urology: 52.0% for both gender groups (Figure 6).

**Figure 7:**
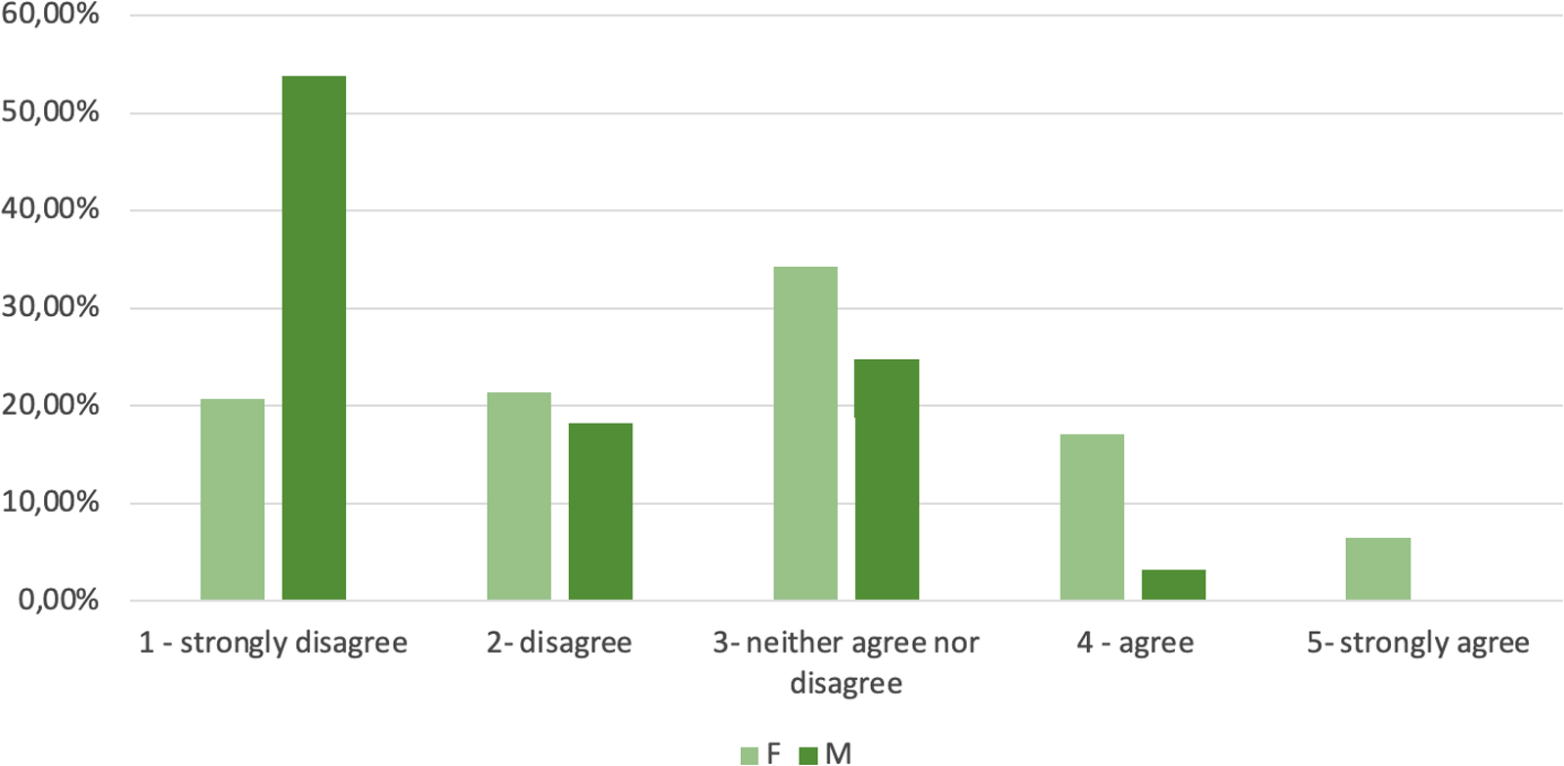
Percentage of Likert value attribution to the statement “Sexist environment” among male and female respondents in the group of respondents not choosing Urology.

The main reasons for medical students in general for not choosing Urology is the lack of interest in the specialty. Interestingly, values ≥4 were assigned in similar proportions by the two gender groups for all statements, with an exception for “Sexist environment”: 3.2% for males and 23.4% for females (*p* < 0.0001) (Figure 6). The different distributions in value attribution to the statement “Sexist environment” between male and female responders are shown in Figure 7.

## Discussion

Despite a historical general conception of doctors being thought of as masculine entities, in the last 40 years, the figure of women in medicine has been consistently arising until reaching unequivocal numerical predominance (4).

The process of feminisation of medicine (13, 14) has also been evident in Italy, as reported by the higher number of enrolled, matriculated, and graduated female medical students compared with their male counterparts.

What happens after graduation?

Our study showed the tendency of Italian female doctors to prefer medical specialties rather than surgical specialties, with the percentage of female matriculants to surgical specialties decreasing through the years. Moreover, there is a clear uneven gender distribution among the different surgical specialties. In fact, women outnumber males in Gynaecology, Thoracic, and Vascular Surgery, whereas males prefer Plastic Surgery, Orthopaedics, and Urology. It also showed that this tendency is strongly related to the perception of surgical specialties as “Sexist environment.”

In Urology, women have always been underrepresented (11), and our study confirms that in Italy, there is still a wide gender gap in this professional field. Therefore, further considerations should be made regarding the tendency of female doctors, who will consider the surgical field of specialisation, not to choose Urology over other surgical specialties.

Choosing a specialty is one of the most important decisions young doctors must face. They undergo an extremely complex decision-making process that involves a variety of factors such as early exposure to the subjects during studies (13), the perspective of a good work–life balance (14, 15), the possible room for a maternity leave (16), and the possibility of being inspired by same sex mentors (17). Gender bias exists among men and women, and female physicians may be subject to negative stereotyping in male-dominated fields, which are more likely to be surgical in nature, thus avoiding specialties like Orthopaedics and Urology (12).

Our survey has investigated the importance that Italian medical students attribute to personal aptitude towards a subject, the perspective of a favourable work-life balance, personal experience during medical school, and the opportunity of a good career and income. The results demonstrate that the main discriminant is passion and, therefore, the personal interest that a specialty sparks in them during classes or internships.

Interestingly, the main reason why medical students do not consider surgery and in particular Urology as a career appears to be related to the little consideration and space that surgical subjects are accorded during medical school, and the subsequent lack of interest into these subjects. This highlights the crucial role of an early exposure of students to surgery and suggesting an implementation of practical and theoretical surgical experiences in the core curriculum of the medical school.

The literature shows the importance of early exposure for medical students to consider Urology as a future specialty (12). In fact, inadequate exposure to Urology and poor staff and resident involvement in undergraduate education were identified as potential causes for misperception of the specialty (12). The roots of male prevalence in Urology may as well arise from the poor knowledge of medical students to this discipline (18), and the consequent misperception of it dealing exclusively with male genitalia (19) instead of encompassing the entire genitourinary system, thus being relevant to both genders. This wrong perception can also give a deeper understanding of the poor attractiveness of the urological field for women.

Moreover, due to the broad spectrum of diseases included under the label of Urology and to the demographic shift that society is facing, leading to a higher prevalence of urological diseases among the elderly population (20), this specialty will be affected negatively by an increase in workload, and the current trend of medical students drifting away from surgery could significantly affect the delivery of urological services.

Our survey has demonstrated that Urology, as all surgical specialties overall, is perceived as a sexist environment, and this impression of it may affect the decision-making process of female medical students more than it affects male students.

Interestingly, while a significant difference between female and male was found regarding “sexist environment” when “Why would you not consider Urology as a career?” was questioned, the same difference did not result when “Why would you consider Urology as a career?” was asked.

The straightforward explanation of this phenomenon might be the existence of a different mindset between women who dismiss Urology because they perceive it as a sexist environment and women who, despite acknowledging the stereotype of the Urological field being a sexist environment, do not discard Urology as an option for their future.

Unconscious gender-based assumptions and stereotypes are deeply embedded in the patterns of thinking of both men and women. This consideration has been extensively proven by the results of the implicit association test (IAT), a measure within social psychology designed to detect a person’s subconscious association between mental representations of concepts in memory. It is frequently used to estimate implicit stereotypes retained by test subjects (21).

The IAT was proven to apply especially to healthcare professionals. In fact, this group shows more relevant implicit associations linking men with career and women with family than professionals from other fields (22). Therefore, men might be viewed as having more “agentic” traits, which include being strong, action oriented, ambitious, and competitive, whereas women might be viewed as having more “communal” traits, which include being gentle, sympathetic, and submissive (13).

The same idea is also used to categorise the different medical specialties: “agentic” specialties are mostly Neurosurgery, Orthopaedics, and Urology, while “communal” specialties are Paediatrics, family medicine, primary care internal medicine specialties, including Geriatrics (23). Such categorisation might underlie a gender-specialty bias as shown in a study enrolling 131 surgeons (in practice and in training) who were administered a modified IAT, the results of which indicated a significant implicit association linking men with surgery and women with family medicine (22).

Moreover, the IAT also represents a useful mean for surgical educators to self-assess personal gender-related biases and was, in fact, included in a list of guidelines proposed by Hemphill et al. (24) to address this issue.

Furthermore, females working in male-dominated surgical fields like Urology may be affected by the risk of “microaggressions” (25) or worse, sexual harrassement, and discrimination (26). The sexist environment that medical students may perceive in this regard could then negatively influence their choices.

In consideration of the results of our survey, suggesting the existence of two groups of women with a different attitude towards male-dominated specialties, and towards Urology, the next research objective would be to study the psychological mechanisms that might determine this different attitude, to understand whether it is related to the distinction between communal and agentic women, and lastly how their characteristics are perceived and judged in Urology.

In conclusion, the tendency to avoid Urology as the specialty of choice might determine an uneven distribution of human resources, skewed towards a very specific subset of gender or mindset, possibly causing a qualitative decline of the provided services. Therefore, it is important to develop strategies to improve medical student intake into Urology to match the projected demand in future and to attract not only an adequate number of doctors, but also the best and most brilliant ones.

## Conclusion

Our study has proven that in Italy the prevalence of female medical graduates does not mirror the proportion of female doctors choosing a career in some surgical specialties, including Urology. While the reasons for this phenomenon remain unclear and influenced by multiple factors, the presence of a gender-biased perception of a sexist environment represents a possible explanation.

## Data Availability

The raw data supporting the conclusions of this article will be made available by the authors, without undue reservation.
