# Corrigendum: Is urology a gender-biased career choice? A survey-based study of the Italian medical students'; perception of specialties

**DOI:** 10.3389/fsurg.2022.1014036

**Published:** 2022-08-30

**Authors:** Sofia Reale, Luca Orecchia, Simona Ippoliti, Simone Pletto, Serena Pastore, Stefano Germani, Alessandra Nardi, Roberto Miano

**Affiliations:** ^1^ Urology Unit, Centre Hospitalier Universitaire Vaudois (CHUV), Lausanne, Switzerland; ^2^ Urology Unit, Policlinico Tor Vergata, Rome, Lazio, Italy; ^3^ Urology Department, Harrogate and District NHS Foundation Trust, Harrogate, United Kingdom; ^4^ Department of Mathematics, University of Rome Tor Vergata, Rome, Lazio, Italy; ^5^ Department of Surgical Sciences, University of Rome Tor Vergata, Rome, Italy

**Keywords:** feminisation of medicine, specialty training, urology training, medical students, sexist environment

A corrigendum on Is urology a gender-biased career choice? A survey-based study of the Italian medical students' perception of specialties By Reale S, Orecchia L, Ippoliti S, Pletto S, Pastore S, Germani S, Nardi A, Miano R. (2022). Front. Surg. 9:962824. doi: 10.3389/fsurg.2022.962824

**Incorrect Author Name**

In the published article, author names were incorrectly written as Reale Sofia, Orecchia Luca, Ippoliti Simona, Pletto Simone, Pastore Serena, Germani Stefano, Nardi Alessandra, Miano Roberto.

The correct order is Sofia Reale, Luca Orecchia, Simona Ippoliti, Simone Pletto, Serena Pastore, Stefano Germani, Alessandra Nardi, Roberto Miano.

**Incorrect Citation**

This error is repeated also in the citation, which states ‘Sofia R, Luca O, Simona I, Simone P, Serena P, Stefano G, Alessandra N, Roberto M (2022) Is Urology a gender-biased career choice? A survey-based study of the Italian medical students' perception of specialties. Front. Surg. 9:962824. doi: 10.3389/fsurg.2022.962824.' The correct author name order is: Reale S, Orecchia L, Ippoliti S, Pletto S, Pastore S, Germani S, Nardi A and Miano R.

This error is repeated also in pages 2 to 9, in the published article top left corner states Sofia et al. The correct spelling is Reale et al.

**Incorrect Copyright Statement**

In the published article, there was an error in the Copyright statement. The statement was incorrectly written as “[© 2022 Sofia, Luca, Simona, Simone, Serena,Stefano, Alessandra and Roberto.]”. The corrected statement is “[©2022, Reale, Orecchia, Ippoliti, Pletto, Pastore, Germani, Nardi and Miano. This is an open-access article distributed under the terms of the Creative Commons Attribution License (CC BY). The use, distribution or reproduction in other forums is permitted, provided the original author(s) and the copyright owner(s) are credited and that the original publication in this journal is cited, in accordance with accepted academic practice. No use, distribution or reproduction is permitted which does not comply with these terms.”

The authors apologize for this error and state that this does not change the scientific conclusions of the article in any way. The original article has been updated.

