# Erratum: Is urology a gender-biased career choice? A survey-based study of the Italian medical students' perception of specialties

**DOI:** 10.3389/fsurg.2023.1178816

**Published:** 2023-03-15

**Authors:** Frontiers Production Office

**Affiliations:** Frontiers Media SA, Lausanne, Switzerland

**Keywords:** feminisation of medicine, specialty training, urology training, medical students, sexist environment

An Erratum on Is Urology a gender-biased career choice? A survey-based study of the Italian medical students' perception of specialties By Reale S, Orecchia L, Ippoliti S, Pletto S, Pastore S, Germani S, Nardi A and Miano R. (2022) Front. Surg. 9:962824. doi: 10.3389/fsurg.2022.962824

An omission to the funding section of the original article was made in error. The following sentence has been added: “Open access funding was provided by the University of Lausanne”.

The original version of this article has been updated.

