# Supplementary material for: Is Urology a gender-biased career choice? A survey-based study of the Italian medical students' perception of specialties
Source: Front Surg. 2022 Jul 29;9:962824. doi: 10.3389/fsurg.2022.962824 (PMC9373042; doi:10.3389/fsurg.2022.962824)
Supplement: Supplementary file 1 [file Table1.docx]

| Medical specialties | Surgical specialties | Services specialties |
| --- | --- | --- |
| Emergency medicine | General surgery | Pathology |
| Geriatrics | Paediatric surgery | Clinical Microbiology |
| Sports medicine | Plastic surgery | Clinical Biochemistry |
| Thermal medicine | Obstetrics & Gynaecology | Radiology |
| Oncology | Orthopaedics | Radiotherapy |
| Community medicine | Urology | Nuclear medicine |
| Allergology and immunology | Maxillo-facial surgery | Anaesthesiology |
| Dermatology | Neurosurgery | Audiology |
| Haematology | Ophthalmology | Physical medicine |
| Endocrinology | ENT | Medical genetics |
| Nutrition sciences | Cardiac surgery | Clinical pharmacology |
| Gastroenterology | Thoracic surgery | Preventive medicine |
| Cardiology | Vascular surgery | Work medicine |
| Pneumology |  | Legal medicine |
| Infectious diseases |  | Clinical statistics |
| Nephrology |  |  |
| Rheumatology |  |  |
| Neurology |  |  |
| Childhood neuropsychiatry |  |  |
| Psychiatry |  |  |
| Paediatrics |  |  |

Tab. 2. List of specialties included in medical, surgical and services area in Italy.
